# Endogenous Semaphorin-7A Impedes Human Lung Fibroblast Differentiation

**DOI:** 10.1371/journal.pone.0170207

**Published:** 2017-01-17

**Authors:** Stephane Esnault, Elizabeth E. Torr, Ksenija Bernau, Mats W. Johansson, Elizabeth A. Kelly, Nathan Sandbo, Nizar N. Jarjour

**Affiliations:** 1 Department of Medicine, Division of Allergy, Pulmonary and Critical Care Medicine, the University of Wisconsin-Madison School of Medicine and Public Health, Madison, Wisconsin, United States of America; 2 Department of Biomolecular Chemistry, the University of Wisconsin-Madison School of Medicine and Public Health, Madison, Wisconsin, United States of America; University of Rochester School of Medicine and Dentistry, UNITED STATES

## Abstract

Semaphorin-7A is a glycosylphosphatidylinositol-anchored protein, initially characterized as an axon guidance protein. Semaphorin-7A also contributes to immune cell regulation and may be an essential pro-fibrotic factor when expressed by non-fibroblast cell types (exogenous). In mouse models, semaphorin-7A was shown to be important for TGF-ß1-induced pulmonary fibrosis characterized by myofibroblast accumulation and extracellular matrix deposition, but the cell-specific role of semaphorin-7A was not examined in fibroblasts. The purpose of this study is to determine semaphorin-7A expression by fibroblasts and to investigate the function of endogenously expressed semaphorin-7A in primary human lung fibroblasts (HLF).

Herein, we show that non-fibrotic HLF expressed high levels of cell surface semaphorin-7A with little dependence on the percentage of serum or recombinant TGF-ß1. Semaphorin-7A siRNA strongly decreased semaphorin-7A mRNA expression and reduced cell surface semaphorin-7A. Reduction of semaphorin-7A induced increased proliferation and migration of non-fibrotic HLF. Also, independent of the presence of TGF-ß1, the decline of semaphorin-7A by siRNA was associated with increased α-smooth muscle actin production and gene expression of periostin, fibronectin, laminin, and serum response factor (SRF), indicating differentiation into a myofibroblast. Conversely, overexpression of semaphorin-7A in the NIH3T3 fibroblast cell line reduced the production of pro-fibrotic markers. The inverse association between semaphorin-7A and pro-fibrotic fibroblast markers was further analyzed using HLF from idiopathic pulmonary fibrosis (IPF) (n = 6) and non-fibrotic (n = 7) lungs. Using these 13 fibroblast lines, we observed that semaphorin-7A and periostin expression were inversely correlated. In conclusion, our study indicates that endogenous semaphorin-7A in HLF plays a role in maintaining fibroblast homeostasis by preventing up-regulation of pro-fibrotic genes. Therefore, endogenous and exogenous semaphorin-7A may have opposite effects on the fibroblast phenotype.

## Introduction

Pulmonary fibrosis is a complication of many chronic respiratory disorders, and is best illustrated in idiopathic pulmonary fibrosis (IPF) [1,2]. Pulmonary fibrosis is characterized by the accumulation of extracellular matrix (ECM)-producing fibroblasts, termed myofibroblasts [3,4]. In addition to producing ECM proteins such as periostin, collagen, and fibronectin, myofibroblasts form robust actin stress fibers, express smooth muscle α-actin (α-SMA) and exhibit enhanced contractile activity similar to smooth muscle cells [5,6]. The change of phenotype from fibroblast into myofibroblast can be induced by transforming growth factor-ß (TGF-ß1), under the control of the SMAD proteins and serum response factor (SRF) [7,8]. Moreover, myofibroblasts display enhanced proliferation, migration, and extracellular matrix formation compared to fibroblasts [9–11].

Semaphorin-7A is part of a family of secreted and membrane-associated proteins, which are involved in both the nervous and immune systems [12]. Semaphorin-7A and its receptor, plexin-C1, have been implicated in neutrophil recruitment into the injured lung [13,14]. Via its other receptors, the α1ß1 or αvß1 integrins, semaphorin-7A stimulates macrophages to either produce pro-inflammatory or anti-inflammatory cytokines, respectively [15,16]. *In vivo*, semaphorin-7A’s role as a pro- or anti-inflammatory protein remains uncertain as two different reports provided opposing conclusions despite using the same experimental autoimmune encephalomyelitis (EAE) model [15,17]. In addition to its role in immunity, several reports have demonstrated the requirement of semaphorin-7A for TGF-ß-induced pulmonary fibrosis. In fact, mice lacking the gene for semaphorin-7A had attenuated pulmonary fibrosis in response to TGF-ß overexpression [18]. In humans, increased semaphorin-7A gene and protein expression have been shown in patients with systemic sclerosis-related interstitial lung disease, IPF and liver fibrosis [19–21]. Taken together, these studies support the pro-fibrotic function of semaphorin-7A. However, the direct effect of semaphorin-7A on the fibroblast phenotype remains uncertain, as previous work in the field has not specifically examined its cell specific role in fibroblasts. Another member of the semaphorin family, semaphorin-3E, inhibits human airway smooth muscle cell proliferation and migration [22]. We have shown that exogenous recombinant semaphorin-7A slightly increased α-SMA production by primary lung fibroblasts [23]. Interestingly, Jongbloets *et al*. [12], have suggested opposing functions for exogenous versus endogenous semaphorin-7A referencing works by Delorme *et al*. [24] and Spencer *et al*. [25]. From these studies it is reasoned that exogenous semaphorin-7A activates osteoblasts whereas endogenous semaphorin-7A may suppress osteoblast differentiation. In the present study, we sought to analyze the expression of semaphorin-7A in cultured HLF and subsequently to determine the role of endogenous semaphorin-7A on the fibroblast phenotype. Some of these results have been reported in abstract form (*Annual Meeting of American Thoracic Society*, *San Francisco 2016*; A4953)

## Materials and Methods

### Isolation and Primary Culture of Human Pulmonary Fibroblasts

**Fig 1:**
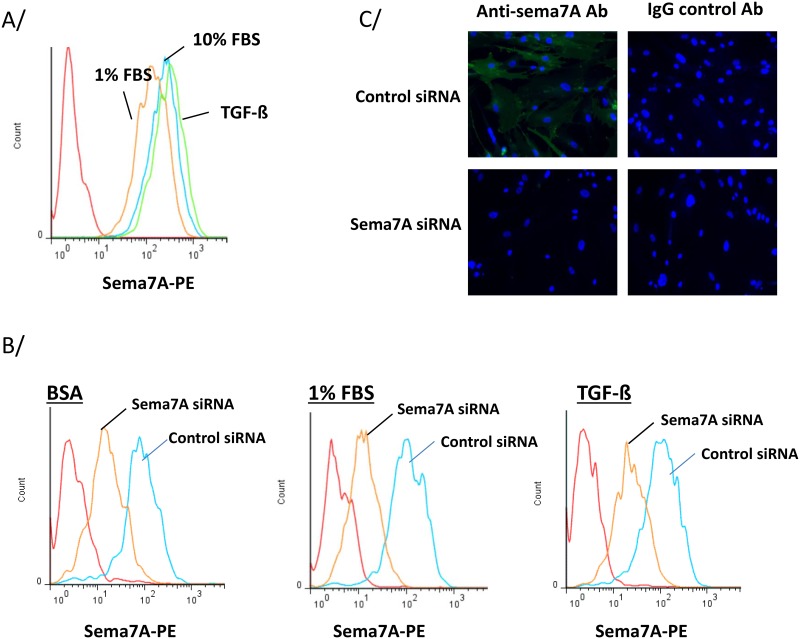
Semaphorin-7A siRNA reduces semaphorin-7A present in non-fibrotic human lung fibroblasts (HLF). A/ and B/ Surface semaphorin-7A was analyzed by flow cytometry. Histograms are representative of 3 experiments. A/ HLF derived from non-fibrotic lungs were cultured in 1% or 10% FBS or TGF-ß1 (1 ng/ml) for 24 h. B/ HLF were cultured and treated with 30 nM of siRNA. After 24 h, cells were cultured in BSA or 1% FBS, or were activated with TGF-ß1 for 24 h. C/ HLF were treated with 30 nM of siRNA for 24 h and cells were stained with a goat anti-semaphorin-7A antibody or a goat IgG control antibody. Immunofluorescent staining was observed with a 20X objective.

**Fig 2:**
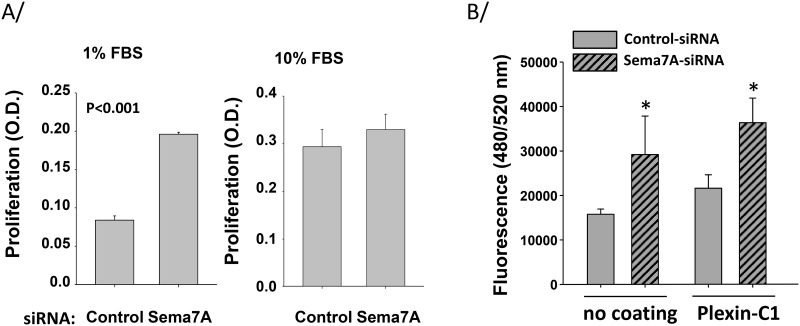
Semaphorin-7A diminishes HLF proliferation and migration. HLF derived from non-fibrotic lungs were treated with either control or semaphorin-7A siRNA and cultured in 1% or 10% FBS for 48 h. A/ BrdU was incubated with cells for 6 h. Absorbance was measured using a spectrophotometric plate reader at dual wavelengths of 450–550 nm. Each condition was performed in quadruplicate (4 wells) and graphs show a mean ± SEM of 3 experiments. B/ Cells were resuspended in 0.1% FBS and migration on plastic or plexin-C1 (10 μg/ml) toward medium with 10% FBS was measured after 20 h. Graph shows an average ± SEM of 4 experiments, and * indicates statistical difference between control-siRNA and sema7A-siRNA-treated HLF.

**Fig 3:**
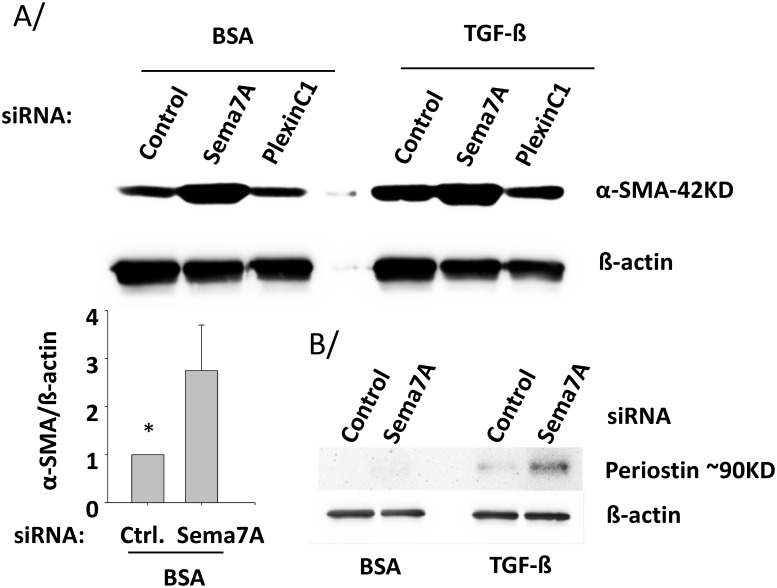
Semaphorin-7A blocks α-SMA and periostin production in HLF. HLF derived from non-fibrotic lungs were treated with the indicated siRNAs for 24 h. HLF were subsequently starved in BSA for 24 h before either adding TGF-ß (1 ng/ml) or left in BSA for 20 h. The amounts of α-SMA and periostin in the cell lysates were determined by western-blot. A/ Graph shown is an average of 3 experiments, and * indicates that control (Ctrl) siRNA-treated HLF produce significantly less α-SMA than sema7A-siRNA-treated HLF. B/ Amount of intracellular periostin is representative of 2 experiments.

**Fig 4:**
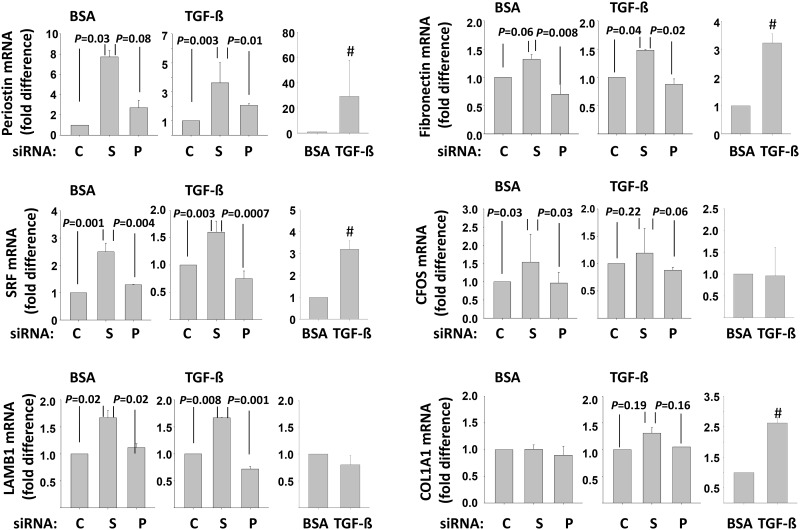
Semaphorin-7A inhibits the expression of myofibroblast markers. Non-fibrotic HLF were treated with control-siRNA (C), sema7A-siRNA (S) or plexin C1-siRNA (P) for 24 h before starvation in BSA for 24 h. Then, the cells were either kept in BSA or activated with TGF-ß (1 ng/ml) for 20 h. Real-time PCR was used to measure the level of expression of the indicated genes. For each gene, the first 2 graphs show the difference between C, S or P treatment, with C fixed at 1. The third graph displays the difference between TGF-ß and BSA after treatment with the control-siRNA. Graphs are an average of 3 experiments. *P* values from ANOVA analyses are shown. # indicates a statistical difference between TGF-ß and BSA.

**Fig 5:**
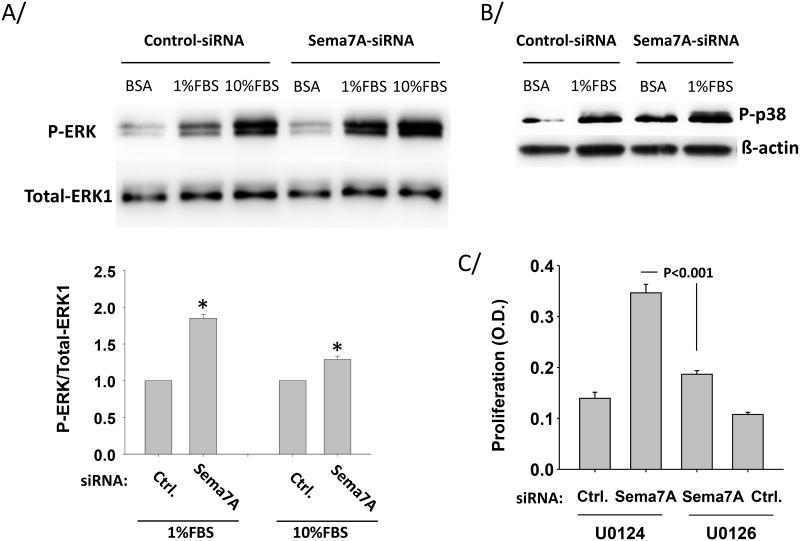
Semaphorin-7A reduces serum-induced ERK and p38 phosphorylation in HLF. **HLF derived from non-fibrotic lungs** were treated with control-siRNA (Ctrl.) or semaphorin7A-siRNA (sema7A) for 24 h before starvation in BSA for 24 h. A/ Then cells were activated with 1% or 10% FBS for 15 min. ERK phosphorylation (P-ERK) was analyzed by western-blot. The graph shows ratios of phospho-ERK versus total ERK1, and is an average of 3 experiments. * indicates that the increase of phospho-ERK after semaphorin-7A (Sema7A)-siRNA-treatment is statistically different from phospho-ERK after control-siRNA (Ctrl) treatment. B/ Cells were activated with 1% FBS for 30 min and a representative blot of 2 experiments shows phosphorylation of p38 (P-p38). C/ Cells were treated with the indicated siRNA and cultured for 48 h in 1% FBS along with a phospho-ERK inhibitor (U0126) or its inactive analog (U0124) during the last 24 h. Proliferation was determined by adding BrdU in the last 6 h of the culture. One representative experiment of 2 using 2 different HLF lines is shown. Average ±SEM of 4 wells is shown.

Human lung fibroblasts (HLF) were isolated as described previously [26] using de-identified tissue samples from thoracic surgical resection or lung explant specimens. These samples were collected and characterized in collaboration with the biobanking services run by the Carbone Cancer Center Translational Science BioCore at the University of Wisconsin-Madison, under Institutional Review Board approval. To obtain non-fibrotic fibroblasts (for Figs 1–5), we utilized lobectomy or biopsy specimens from patients undergoing lung resection and who did not have interstitial lung disease. All specimens were examined by a pathologist to confirm that no underlying parenchymal lung disease (emphysema, idiopathic lung diseases, etc.) was present on histology. Fibrotic lung tissues were obtained from either biopsy specimens from patients with idiopathic pulmonary fibrosis (IPF) or from lung explants at the time of lung transplant for IPF. All fibrotic tissues were confirmed by a pathologist to have a usual interstitial pneumonia (UIP) pattern on histology.

To isolate fibroblasts, tissue specimens were placed in DMEM with 100 units/ml streptomycin, 250 ng/ml amphotericin B, 100 units/ml penicillin, and 10 μg/ml ciprofloxacin. Alveolated lung tissue was minced and plated onto 10-cm plates in growth medium containing DMEM supplemented with 10% FBS, 2 mM L-glutamine, and antibiotics as above. Expanded populations of fibroblasts were subsequently subcultured after 4–5 days, resulting in the development of a homogenous fibroblast population. All primary cultures were used from passages 5–10 and maintained on tissue culture plastic until the time of experiments. Two to three different HLF lines from different donors were used for each experiment. For Fig 7, HLF were prepared from lungs obtained from 6 patients with IPF and 7 subjects without pulmonary fibrosis. HLF were cultured in DMEM/10% FBS before RNA extraction.

### siRNA Knockdown

Cells were seeded at 5x10^4^ cells/ml 24 h before transfection, reaching 70–80% confluency at the time of transfection. A range of concentrations of siRNA was tested and 30 nM were selected for this study according to its inhibitory effect on surface semaphorin-7A. siRNA (Santa Cruz Biotechnology, Santa Cruz, CA) was transfected using RNAiMAX transfection reagent (LifeTechnologies, Eugene, OR) diluted in Opti-MEM (Gibco LifeTechnologies) with 1μl of RNAiMAX per 10 pmol of siRNA according to the manufacturer’s instructions. Cells were cultured in 10% FBS for 24 h and then either maintained in 10% FBS or were serum-starved (1% FBS or 0.1% Bovine serum albumin) before stimulation with TGF-ß1 (EMD Biosciences, Gibbstown, NJ; 1ng/ml) for 24 or 48h.

### Flow Cytometry

After the indicated culture conditions and treatments, HLF or NIH3T3 cells were detached using enzyme-free cell dissociation solution (Sigma-Aldrich, St. Louis, MO) supplemented with 10 mM EDTA. Cells were then stained with either PE-conjugated anti-CD108 (semaphorin-7A) or an isotype control (BD Biosciences, San Jose, CA). Flow histograms were generated using FlowJo analysis software (FlowJo, Ashland, OR).

### Immunofluorescent Staining

HLF were treated with either control or semaphorin-7A siRNA and cultured for 24h. Cells were subsequently plated into 8-well chamber-slides (Ibidi, Munich, Germany) at 1.65x10^5^ per ml and allowed to adhere for 24 h. The cells were washed twice with TBS, fixed with 4% paraformaldehyde/TBS for 30 min at room temperature (RT) and permeabilized with 0.2% Triton X-100/TBS (Thermo Fisher Scientific, Rockford, IL) for 5 min at RT. Cells were then blocked with 10% BSA in TBS for 1h at RT and incubated overnight at 4°C with the relevant primary antibody (1.3μg/ml in 2% BSA/0.2% Triton X-100/TBS). Cells were washed with TBS, incubated with secondary antibody in 2% BSA/TBS for 75 min at 37°C, washed with TBS, incubated with DAPI/TBS (0.5μg/ml) for 10 min RT, washed with TBS and mounted in Ibidi mounting media (Munich, Germany). Goat polyclonal anti—semaphorin-7A Ab and goat IgG control were from R&D Systems (Minneapolis, MN). Secondary Alexa Fluor-488 donkey anti goat antibody was from Life Technologies (Eugene, OR). Immunofluorescent images were obtained using an Olympus 1X71 fluorescent microscope and Q imaging Retiga 2000R camera.

### Reverse Transcription Quantitative Real Time PCR

Total RNA was extracted from cells line using the RNeasy Mini Kit (Qiagen, Valencia, CA). The reverse transcription reaction was performed using the Superscript III system (Invitrogen/Life Technologies, Grand Island, NY). mRNA expression was determined by real-time PCR (RT-PCR) using SYBR Green Master Mix (SABiosciences, Frederick, MD), and specific primers (S1 Table) designed using Primer Express 3.0 (Applied Biosystems, Carlsbad, CA) and blasted against the human genome to determine specificity using http://www.ncbi.nlm.nih.gov/tools/primer-blast. Human COLA1A1, mouse periostin, fibronectin and α-SMA primers and the TaqMan hydrolysis probe were obtained from Applied Biosystems (S1 Table). The reference gene, ß-glucuronidase (GUSB), forward: CAGGACCTGCGCACAAGAG, reverse: TCGCACAGCTGGGGTAAG), was used to normalize the samples. Applied Biosystems (ABI/Invitrogen, Carlsbad, CA) 7500 Sequence detector was used. Standard curves were performed and efficiencies were determined for each set of primers. Efficiencies ranged between 93 and 96%. Data are expressed as fold difference using the comparative cycle threshold (ΔΔCT) method as described previously [23].

### Proliferation, Migration and Adhesion

To measure HLF proliferation, cells were plated into wells of a 96 well plate at 2x10^3^ cells per well in 100 μl DMEM 10% FBS and left to adhere overnight. 10 μl of the appropriate siRNA (30 nM) was added to the relevant wells with DMEM 10% FBS. After 24 h cells were incubated in 10 or 1% FBS and incubated for 48 h at 37°C. The ERK inhibitor (U0126; 10μg/ml) or its inactive analog (U0124; 10μg/ml) were added 24 h after cells were changed into 1% FBS medium, and cells were cultured for another 24 h. BrdU cell proliferation assay (Calbiochem, EDM Millipore Corp., CA) was performed as directed by the manufacturer. BrdU was incubated with cells for 6 h. Absorbance was measured using a spectrophotometric plate reader at dual wavelengths of 450–550 nM. Each condition was performed in quadruplicate (4 wells) and the controls, no cell and no BrdU were deducted from the measured absorbance.

Migration of HLF was measured using QCM^™^ 24-well fluorometric cell migration assay with an 8 μm pore size well insert (EMD, Millipore Corp.). HLF were plated into a 6-well plate at 1x10^5^ cells per well in DMEM 10% FBS and left to adhere overnight. Then cells were treated with 30 nM siRNA for 2 days in 10% FBS. 24-well inserts were left uncoated or were coated with 100 μl of a recombinant human plexin-C1 (10 μg/ml) (R&D Systems) at 37°C for 1 h, and were air-dried overnight under sterile conditions. HLF were detached using enzyme-free cell dissociation solution (Sigma) supplemented with 10 mM EDTA and resuspended at 0.5x10^6^ cells/ml in DMEM 0.1%FBS. 300 μl of the cell suspension was added to each insert, and 500 μl of DMEM 10% FBS was added in the lower chamber. The plate was incubated at 37°C for 20 h. Quantification of the migrated cells was performed as indicated by the manufacturer and fluorescence was measured in a 96-well plate with a fluorescence plate reader using 480/520 nm filter set.

For the adhesion assay, HLF were plated in 10% FBS into wells of a 6-well plate at 1x10^5^ cells per well in DMEM 10% FBS and left to adhere overnight. Cells were treated with siRNA for 2 days in 1% FBS, harvested and 2x10^4^ cells/ml were resuspended in DMEM and 0.1% BSA. A 96-well plate (Costar, Corning, NY) was coated, or not, with 50 μl of plasma fibronectin (20 μg/ml in PBS; Calbiochem) overnight and wells were then saturated with 3% BSA for 1 h at 37°C. 100 μl of cells were added to each well for 30 min with each condition performed in quadruplicate. The plate was shaken at 2000 rpm for 10 s, washed 3 times with 0.1% BSA and cells were fixed with 4% paraformaldehyde for 10 min. The plate was then washed with PBS; cells were stained with crystal violet for 10 min, washed in H_2_O, dried and lysed with 2% SDS (30 min at RT). Absorbance was measured by a spectrophotometric plate reader at 550 nM. No cell control was deducted from the measured absorbance.

### Western-Blot

Cells were lysed in an assay buffer containing 25 mm HEPES (pH 7.5), 150 mm NaCl, 1% Triton X-100, 0.1% SDS, 2 mm EDTA, 2 mm EGTA, 10% glycerol, 1 mm NaF, 200 μm sodium orthovanadate, and protease inhibitor mixture (Sigma), or directly in Laemmli buffer (10% SDS) before boiling for 5 min and loading onto a 10% SDS-PAGE gel. Immunoblot analysis was performed as previously described [11,27]. Mouse monoclonal antibodies against β-actin and smooth muscle-α-actin (α-SMA) were from Sigma. Monoclonal anti-periostin mAb Stiny-1 was from Adipogen (San Diego, CA). Rabbit anti-phospho-ERK1/2 and phospho-SMAD2 antibodies were from Cell Signaling (Danvers, MA). The rabbit anti-phospho-p38 antibody was from Genetel Laboratories (Madison, WI). The mouse monoclonal antibody anti-ERK1 was from Transduction Laboratories (Lexington, KY). The rabbit polyclonal anti-mouse Semaphorin7A antibody used on NIH3T3 cell lysates was from Abgent (San Diego, CA). Rabbit polyclonal antibody against total mouse fibronectin was from Abcam (Cambridge, MA). Mouse monoclonal antibody against GAPDH was from Santa Cruz Biotech. Secondary HRP-conjugated anti-rabbit IgG Ab and anti-mouse IgG Ab were from Pierce/Thermo Fisher Scientific (Rockford, IL) (anti-rabbit), from Amersham-GE Healthcare Life Sciences (Chicago, IL) or from Calbiochem (anti-mouse). Immunoreactive bands were visualized with Super Signal West Femto chemiluminescent substrate (Pierce/Thermo Fisher Scientific) or Amersham ECL Prime Western Blotting Reagent (GE Healthcare). Bands were quantified using the FluorChem Q Imaging System (Alpha Innotech/ProteinSimple, Santa Clara, CA) or digital chemiluminescent images were taken by a GE LAS4000 chemiluminescence imager. Densitometry of selected blots was performed using ImageGuage software (GE Healthcare) or Image J.

### DNA Transfection

Expression of human semaphorin-7A in NIH3T3 was achieved using the Expression-Ready cDNA clone for semaphorin-7A in pTCN (TransOMIC Technologies, Huntsville, AL).

Transient DNA transfections were performed using GenJet reagent (SL100488, SignaGen Laboratories, Gaithersburg, MD) following the standard manufacturer's instructions. Cells were transfected with either 750 ng of pcDNA3.1 or 750 ng of pTCN-SEMA7A. Cells were placed in growth medium overnight and then serum-starved for 24 h followed by stimulation with TGF-ß (1 ng/ml) for 24 or 48 h.

### Statistical Analysis

For Fig 4 and S4 Fig, mixed effect ANOVA with fixed effect term for treatment and random effect term for variation between experiments was performed. For Fig 7, gene expression levels (fold difference) were log transformed and the Pearson coefficient was used. The *t* test was used for the comparison of 2 groups. Correlation and *t* tests were performed using using SigmaPlot 11.0 software package.

## Results

We first examined the expression of semaphorin-7A on the cell surface of non-fibrotic HLF by flow cytometry. Fig 1A shows high staining for surface semaphorin-7A in HLF cultured in 1% FBS, 10% FBS or with TGF-ß1. In order to begin analyzing the function of endogenous semaphorin-7A on fibroblasts, we reduced the production of semaphorin-7A in fibroblasts using semaphorin-7A-siRNA. The efficacy of the semaphorin-7A-siRNA on its target is shown on S1 Fig. The specific level of gene expression reduction was compared to an irrelevant control-siRNA provided by the manufacturer and to the effect on the non-targeted semaphorin-7A receptor, plexin C1. The semaphorin-7A-siRNA reduced the level of semaphorin-7A mRNA by ~90% and had no effect on plexin-C1 mRNA. Importantly, semaphorin-7A-siRNA reduced HLF semaphorin-7A protein as determined by flow cytometry (Fig 1B) and the diffuse membrane staining seen by immunocytochemistry (Fig 1C). Immunocytochemistry also demonstrated localization of semaphorin-7A to the cell edge in some cells, including structures resembling filipodia and the lamellipodia (semaphorin-7A in green, S2 Fig).

Remarkably, reduction of endogenous semaphorin-7A in non-fibrotic HLF led to increased proliferation in a suboptimal proliferation condition (1% FBS) (Fig 2A). This was not observed when HLF were cultured in a more optimal condition (10% FBS) (Fig 2A). Thus, semaphorin-7A may restrain fibroblast proliferation under certain conditions. Furthermore, we recently reported that eosinophils produce surface semaphorin-7A and adhere on plexin-C1 [23]. Thus, we assessed how loss of semaphorin-7A modified fibroblast migration on plexin-C1 coated plates. Unexpectedly, reduction of semaphorin-7A led to increased fibroblast migration on plexin C1-coated plates (Fig 2B). Similar to the results on plexin C1-coated plates, migration was also potentiated by semaphorin-7A knockdown on tissue culture plastic, suggesting that this effect was independent of interactions between semaphorin-7A and plexin-C1 (Fig 2B). Of note, these fluctuations in migration were not due to changes in levels of fibroblast adhesion (S3 Fig). These data suggest that semaphorin-7A on fibroblasts modulates their capacity for migration, independent of semaphorin-7A-plexin C1 interactions. Thus, it is possible that semaphorin-7A interacts with ß1 integrins rather than plexin C1 to modify this functional effect, as previously demonstrated in several different models [15,28].

The fibroblast differentiation, into a myofibroblast, phenotype is associated with expression of α-SMA, which increases contractility of fibroblasts leading to wound contraction and augmented matrix remodeling capacity [6,11]. Fig 3A shows that reduction of endogenous semaphorin-7A shifted non-fibrotic HLF toward a myofibroblast phenotype, as determined by α-SMA expression, in the presence or absence of exogenous TGF-ß. Likewise, cellular periostin protein was also detected and was increased by the reduction of semaphorin-7A in TGF-ß-activated HLF (Fig 3B). This suggested that semaphorin-7A expression was inhibiting myofibroblast differentiation. We hypothesized that cell-cell interactions mediated by semaphorin-7A and plexin C1 may account for these findings. Based upon this, we tested whether loss of plexin C1 would mimic the effect of semaphorin-7A knockdown. To do so, we utilized siRNA-mediated knockdown of plexin C1. As shown in S1, plexin-C1 siRNA decreased endogenous plexin-C1 mRNA levels by ~80%, while having no effect on semaphorin-7A mRNA. Plexin-C1-siRNA did not affect α-SMA levels indicating that the effect of endogenous semaphorin-7A on α-SMA levels was independent of endogenous plexin-C1 (Fig 3A).

Additionally, expression of a group of genes that are related to myofibroblast differentiation was analyzed by real-time PCR. The decrease in endogenous semaphorin-7A led to enhanced expression of periostin, fibronectin, the beta 1 chain (LAMB1) of laminins and SRF in a TGF-ß1-independent manner (Fig 4). There was a trend for increased expression of COL1A1 in a TGF-ß1-dependent manner but it did not reach statistical significance (Fig 4). The reduction of plexin-C1 had no significant effects on these genes. With the exception of LAMB1 and CFOS, all of the myofibroblast markers were enhanced by TGF-ß stimulation (Fig 4). Altogether, these results indicate that endogenous semaphorin-7A is critical to limit the differentiation of fibroblasts toward myofibroblasts. Notably, for the pro-fibrotic cytokines, TGF-ß and IL-6, there were trends to slight increases in mRNA expression levels in semaphorin-7A-siRNA-treated HLF, although changes did not reach statistical significance (S4 Fig).

Due to the observed effects on fibroblast proliferation and differentiation, we sought to determine how endogenous semaphorin-7A may alter signaling in HLF. Under treatment with 1% or 10% FBS (conditions that support fibroblast proliferation as shown in Fig 2A), loss of semaphorin-7A resulted in increased ERK1/2 phosphorylation, (Fig 5A). Interestingly, loss of semaphorin-7A under conditions of 1% FBS had a greater effect on ERK1/2 phosphorylation than loss of semaphorin-7A under stimulation with 10% FBS. This may be due to the fact that ERK1/2 signaling is likely maximal with 10% FBS stimulation. We also observed that another mitogen activated protein kinase substrate, p38, was also increased with loss of semaphorin-7A (Fig 5B). We noted that the induction of ERK1/2 phosphorylation with semaphorin-7A knockdown and 1% FBS stimulation correlated well with the induction in proliferation under this condition. Because of these data, and the well-known role of ERK1/2 signaling to control proliferative signaling in cells [29], including fibroblasts [30], we examined its role in potentiating proliferation under conditions where semaphorin-7A was lost. In Fig 5C, we found that ERK blockade with the small molecule inhibitor U0126 reduced semaphorin-7A-siRNA-induced proliferation by >80% compared to its inactive analog. This demonstrates that loss of semaphorin-7A under sub-maximal proliferative conditions (1%FBS) results in induction of fibroblast proliferation via induction of ERK signaling. In this fashion, it is possible that semaphorin-7A may attenuate ERK signaling to prevent proliferation in HLF.

Given the induction of pro-fibrotic markers in semaphorin-7a-siRNA-treated HLF (Figs 3 and 4), we hypothesized that perhaps loss of semaphorin-7A also modified TGF-β-dependent signaling. We examined TGF-β-receptor-associated SMAD phosphorylation using Western blotting for phosphorylated SMAD2 at serine 465/467 as we have previously [8]. SMAD2 remained unphosphorylated under conditions of 1% or 10% FBS stimulation or under conditions where semaphorin-7A was knocked down via siRNA (data not shown). This suggests that semaphorin-7A does not disrupt SMAD signaling.

**Fig 6:**
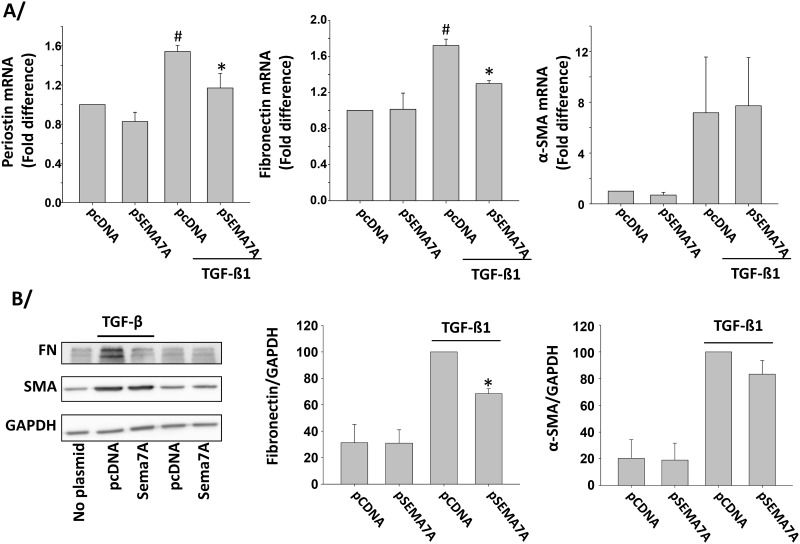
Production of semaphorin-7A in the NIH3T3 fibroblast cell line decreases expression of periostin and fibronectin. The NIH3T3 fibroblast cell line was transfected with 0.75 μg of plasmid control (pcDNA) or semaphorin-7A-expressing plasmid (pSEMA7A) for 24h. Cells were subsequently starved for 24 h and then activated with TGF-ß1 (1ng/ml) or left untreated for 24 h. A/ The indicated genes were analyzed by real-time PCR. Graphs show an average of 3 experiments. # indicates that expression level is increased compared to non-activated (no TGF-ß1) cells and transfected with pcDNA. * indicates that gene expression level was significantly decreased compared to TGF-ß1-activated cells and transfected with pcDNA. B/ The indicated proteins were analyzed by western-blot. A representative blot is shown on the left. Graphs show an average ±SEM of 3 experiments, and * indicates that semaphorin-7A production reduced fibronectin in TGF-ß-activated cells (p = 0.001).

To further analyze the function of endogenous semaphorin-7A in fibroblasts, we attempted to overexpress semaphorin-7A in HLF. However, primary cultures of HLF have a very low transfection rate (data not shown). We therefore chose to use the mouse fibroblast cell line, NIH3T3, due to: 1) the ability to efficiently express semaphorin-7A by transient transfection, 2) the low basal expression of semaphorin-7A so as to allow us to compare the effects of overexpression of semaphorin-7A, and 3) the responsiveness of this cell line to TGF-β and the established use of this cell line in evaluating molecular mechanisms involved in pulmonary fibrosis [31–35]. In contrast to primary HLF semaphorin-7A was not detected by Western-blot in the NIH3T3 cells line (S5A Fig), but was markedly present on the cell surface after transfection with a human semaphorin-7A-expression plasmid (S5B Fig). In this cell line, expression of human semaphorin-7A resulted in reduction of TGF-ß1-induced periostin and fibronectin although α-SMA was not affected (Fig 6A). In addition, the induction of fibronectin protein by TGF-ß was also inhibited by the expression of semaphorin-7A while α-SMA protein did not significantly change (Fig 6B). This further supports the inhibitory characteristic of endogenous semaphorin-7A on markers of differentiation in the fibroblast, and suggests that semaphorin-7A differentially controls the production of ECM and α-SMA.

**Fig 7:**
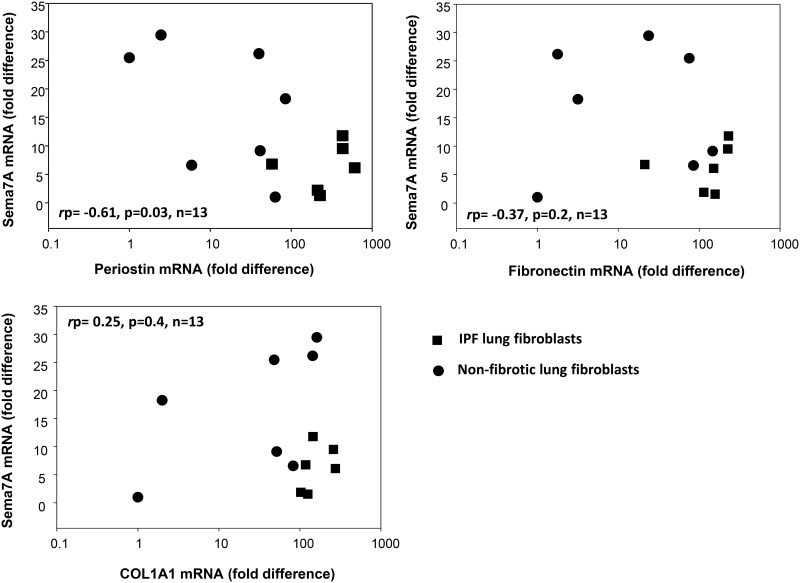
Semaphorin-7A expression is inversely correlated with periostin expression in HLF. Expression of semaphorin-7A (sema7A), periostin, fibronectin and collagen-1 (COL1A1) were measured by real-time PCR in fibroblasts from non-fibrotic (●, n = 7) and IPF lung (■, n = 6). Semaphorin-7A expression was inversely associated with periostin expression level (*r*p = -0.608, *p* = 0.027, n = 13).

Our data, above, suggests an inverse association between semaphorin-7A and myofibroblast molecular markers. To address this question, semaphorin-7A expression level was analyzed vis-à-vis expression of a myofibroblast marker, in HLF, from fibrotic (IPF; n = 6) and non-fibrotic (non-IPF; n = 7) lungs. Periostin was chosen to be analyzed alongside semaphorin-7A due to its essential role in myofibroblast differentiation, tissue remodeling, and lung fibrosis [36–39], and due to its high increased expression level in semaphorin-7A-siRNA-treated HLF (Fig 4). As expected, we observed large variations of periostin expression with higher levels in a “fibrotic” (IPF) origin of HLF compared to a “normal lung” (non-IPF) origin of HLF (*p* = 0.005). Interestingly, analysis of the correlation between semaphorin-7A and periostin expression, demonstrates an inverse correlation between the expression level of these 2 genes (*r*p = -0.61, *p* = 0.03, n = 13, Fig 7). In addition to periostin, fibronectin and COL1A1 expression levels were analyzed vis-à-vis semaphorin-7A. As shown in Fig 7, correlation analysis of fibronectin with semaphorin-7A had a comparable profile to periostin, but the inverse correlation did not reach statistical significance, at least partially due to an outlier fibroblast line, where expression levels were low for both semaphorin-7A and fibronectin (Fig 7). However, in agreement with Fig 4, COL1A1 did not show any association with semaphorin-7A expression (Fig 7). In summary, these data confirm that semaphorin-7A expression is inversely correlated with the expression of the key pro-fibrotic gene, periostin.

## Discussion

Fibroblast proliferation, migration and differentiation constitute major hallmarks of tissue fibrosis. In this study, we identify endogenous semaphorin-7A as a constrainer of these pro-fibrotic characteristics, and an inhibitor of the production of the myofibroblast differentiation markers α-SMA, periostin and fibronectin. In contrast to other studies, semaphorin-7A-mediated effects were independent of exogenous TGF-ß.

We found that primary human lung fibroblasts cultured *in vitro* displayed high levels of semaphorin-7A on their surface. This is in agreement with several studies that have shown localization of semaphorin-7A on fibroblasts and fibrocytes [19,21,28]. The high level of surface semaphorin-7A on HLF allowed us to analyze the effect of endogenous semaphorin-7A reduction on the fibroblast. The reduction of semaphorin-7A in HLF using siRNA led to increased expression of the myofibroblast markers: periostin, α-SMA, SRF, and fibronectin. In addition, we observed that lung fibroblasts which highly expressed periostin had the lowest expression of semaphorin-7A. Taken together, these data support a reciprocal relationship between the expression of fibrotic genes and semphorin-7A expression. Therefore, previous work reporting semaphorin-7A up-regulation in fibrotic tissue may reflect enrichment in the number of fibroblasts and macrophages rather than up-regulation of semaphorin-7A per fibroblast [21], or it may reflect regulation of semaphorin-7A expression in cells that are not fibroblasts. In fact, analyses of lung tissue from IPF patients found semaphorin-7A on airway macrophages and T cells rather than on fibroblasts [18,20]. Nevertheless, the inverse correlation between periostin and semaphorin-7A expression observed in our study suggests that semaphorin-7A is linked to an anti-fibrotic mechanism in fibroblasts. A limitation of our work is that we cannot fully rule out that the fibroblast phenotype from IPF subjects could have changed during the culture *in vitro*. Further studies with immunohistochemical staining may help resolve this issue. Despite these limitations, it should be noted that a protective role has been proposed by Leifkauf *et al*. for semaphorin-7A in the chlorine-induced lung injury model [40].

Interestingly, the shift toward a myofibroblast phenotype happened regardless of whether TGF-ß was present or absent. The lack of requirement for TGF-ß was surprising given a previous report, which describes semaphorin-7A as a requirement for TGF-ß-induced pulmonary fibrosis [18] in a mouse model. However, this study used a germline knockout of semaphorin-7A, which did not allow determination of cell-specific effects. A conditional knockout model targeting fibroblasts would be useful to conclusively determine the role of fibroblast semaphorin-7A on lung or liver tissue fibrosis. Also, the observations of increased tissue semaphorin-7A during fibrosis in humans do not demonstrate a cause-effect relationship between semaphorin-7A and fibroblast behavior.

The differences among studies could be reconciled if exogenous versus endogenous semaphorin-7A had opposite effects on fibrosis. Exogenous semaphorin-7A present on the surface of airway macrophages or activated CD4+ T cells in fibrotic tissues [18,20] may indeed change fibroblasts into a pro-fibrotic phenotype by interacting with fibroblasts via either plexin-C1 or the α1ß1 integrin [15,41]. We have recently shown that recombinant semaphorin-7A could slightly up-regulate α-SMA in HLF [23]. The dichotomy between exogenous and endogenous semaphorin-7A has been reported for osteoblasts. While exogenous semaphorin-7A activates the osteoblast, endogenous semaphorin-7A appears to be an inhibitor of their differentiation [12], supporting differential effects on cells depending on the localization of the protein. Interestingly, semaphorin-7A present on activated T lymphocytes stimulates monocytes to produce pro-inflammatory cytokines, while endogenous semaphorin-7A negatively regulates T lymphocyte activation [15,17].

In addition to its participation in dampening myofibroblast differentiation, we show that loss of semaphorin-7A in fibroblasts permits increased migration. Previous reports suggested that semaphorin-7A-mediated cell migration occurs via surface expression of counter-receptors (ß1-integrins and plexin-C1) for semaphorin-7A. In mouse models of lung injury, neutrophils express plexin-C1, interact with and are guided to the lung by semaphorin-7A, which is expressed by endothelial and epithelial cells [14]. Also, melanocytes express plexin-C1 and ß1-integrins that interact with semaphorin-7A present on the surface of fibroblasts and keratinocytes. Binding of melanocytes to semaphorin-7A via ß1-integrins leads to spreading and dendricity whereas binding via the plexin-C1 receptor has an opposite effect and inhibits the progression of melanoma [28,42]. Dendritic cells also produce surface plexin-C1, which interacts with the viral semaphorin A39R and inhibits cell adhesion and migration via actin cytoskeleton rearrangement [43]. In these studies, semaphorin-7A was acting as an exogenous ligand to plexin-C1 or ß1 integrins present on dendritic cells or melanocytes. Our studies suggest that plexin C1 is not likely to be involved in the dampening effect of endogenous semaphorin-7A on migration. We surmise that semaphorin-7A interactions with β1-integrin may account for these effects, although it is interesting to note that we did not see differences in adhesion upon loss of semaphorin-7A expression. It is also interesting to note that similar to the fibroblasts in our study, van Rijn *et al*. demonstrated that mature dendritic cells produce high level of semaphorin-7A [44]. However, contrary to HLF, reduction of semaphorin-7A by siRNA led to reduced dendritic cell adhesion and migration probably via impaired formation of actin-based protrusions [44]. The relationship between endogenous semaphorin-7A and the proteins of the cytoskeleton is intriguing. We observed by immunocytochemistry that some fibroblasts had intense semaphorin-7A staining localized to cell edges, possibly in the filipodia and the lamellipodia, which are implicated in the elongation of the cells during migration. However, these preliminary observations will require follow-up investigations to determine whether there is a possible relationship between semaphorin-7A expression and actin cytoskeletal organization.

Semaphorin-7A lacks a cytoplasmic tail and therefore it is reasonable to postulate that it interacts with membrane protein partners to regulate cell function. Our study indicates that plexin-C1 is not involved in semaphorin-7A down-regulation of the pro-fibrotic markers. While semaphorin-7A regulated constitutive gene and protein expression in HLF, it also modulated induced p38 and ERK signaling. p38 is known to be required for the induction of myofibroblast differentiation by TGF-β [45]. Likewise, ERK activation by mechanical force was found to be essential for the induction of a myofibroblast phenotype [46]. Conversely, a study by Allegra *et al*., has shown that epithelial-to-mesenchymal transition was inhibited by the activation of the Ras/ERK pathway due at least partially to the regulation of semaphorin-7A expression [47]. This demonstrates a close association between semaphorin-7A and the MAPK pathway. While ERK phosphorylation regulates semaphorin-7A production in epithelial cells, our study indicates that semaphorin-7A controls ERK phosphorylation in fibroblasts. We show here that the effect of semaphorin-7A depletion on ERK phosphorylation explains the observed increase in the proliferative capacity of HLF under sub-maximal serum stimulation. Future studies of the control of the intracellular signaling under quiescent conditions will help our understanding of how semaphorin-7A down-regulates the constitutive expression of the fibrotic markers shown in our study. Likewise, it is possible that cleavage of semaphorin-7A on the cell surface could be important for some of the cell intrinsic effects we observed with respect to phenotypic shift and cell signaling.

In conclusion, our study establishes that expression levels of semaphorin-7A inversely associate with several pro-fibrotic genes in HLF. This finding contrasts with previous studies that have supported the role of semaphorin-7A as a pro-fibrotic molecule, and indicate that therapies targeting semaphorin-7A may not attenuate fibroblast differentiation to a pro-fibrotic myofibroblast. Careful consideration must be made to determine if a therapy reducing semaphorin-7A functions in human fibrosis will result in a beneficial clinical outcome.

## Supporting Information

S1 Fig Semaphorin-7A and plexin-C1 siRNA specifically decreases their respective mRNA target. HLF were treated with siRNA (30 nM) for 48 h, and semaphorin-7A or plexin-C1 mRNA expression levels were analyzed by real-time PCR. Five and 3 experiments were analyzed for semaphorin-7A and plexin-C1, respectively. * indicates mRNA is decreased compared to the other siRNAs, with *p*<0.05.
(PDF)

S2 Fig Semaphorin-7A is present in structure resembling filipodia and lamellipodia. HLF were stained with a goat anti-semaphorin-7A antibody or a goat IgG control antibody. Immunofluorescent staining was observed with a 20x (left panel) or 60X objective (right panel). In all fibroblasts, diffuse membrane staining was apparent (left panel). In some fibroblasts, staining appeared to show localization of semaphorin-7A to cell edges, including structures resembling filipodia and lamellipodia.
(PDF)

S3 Fig Semaphorin-7A has no effect on HLF adhesion. HLF were treated with the indicated siRNA for 48 h. HLF were resuspended in 0.1% BSA and were cultured for 30 min in plastic wells coated or not with fibronectin. The number of adherent cells was evaluated by crystal violet staining and optical absorption at 550 nm. Each condition was performed in quadruplicate and the graph shown is representative of two experiments.
(PDF)

S4 Fig Profibrotic cytokine mRNA expression levels in Semaphorin-7A-siRNA-treated HLF. HLF were treated with control-siRNA (C), sema7A-siRNA (S) or plexin C1-siRNA (P) for 24 h before starvation in BSA for 24 h. Then, the cells were either kept in BSA or activated with TGF-ß (1 ng/ml) for 20 h. Real-time PCR was used to measure the level of expression of the indicated genes. For each gene, the first 2 graphs show the difference between C, S or P treatment, with C fixed at 1. The third graph displays the difference between TGF-ß and BSA after treatment with the control-siRNA. Graphs are an average of 3 experiments. *P* values from ANOVA analyses are shown. # indicates a statistical difference between TGF-ß and BSA.
(PDF)

S5 Fig Expression of transfected human semaphorin-7A the NIH3T3 fibroblast cell line. A/ The analysis of semaphorin-7A in the NIH3T3 fibroblast cell line by western-blot using an anti-human/mouse semaphorin-7A antibody, shows lack of semaphorin-7A in the cell line. Fresh eosinophils (EOS) were used as a positive control. B/ The expression of human semaphorin-7A in the NIH3T3 cell line was greatly induced after transfection with a semaphorin-7A expression vector. Flow cytometry analyses indicate that 0.75 μg of plasmid is optimal for expression of semaphorin-7A on the cell surface.
(PDF)

S1 Table Primer sequences used for real-time PCR. (DOCX)

## Abbreviations

ECM: extracellular matrix
HLF: primary human lung fibroblasts
IPF: idiopathic pulmonary fibrosis
SRF: serum response factor
TGF-ß: transforming growth factor-ß
α-SMA: smooth muscle α-actin
