# Supplementary figures and images for: Endogenous Semaphorin-7A Impedes Human Lung Fibroblast Differentiation
Source: PLoS One. 2017 Jan 17;12(1):e0170207. doi: 10.1371/journal.pone.0170207 (PMC5240965; doi:10.1371/journal.pone.0170207)

Corrected

Figure S1

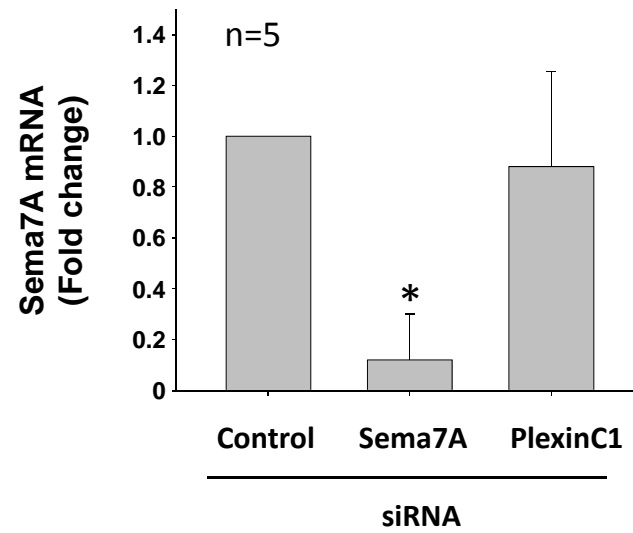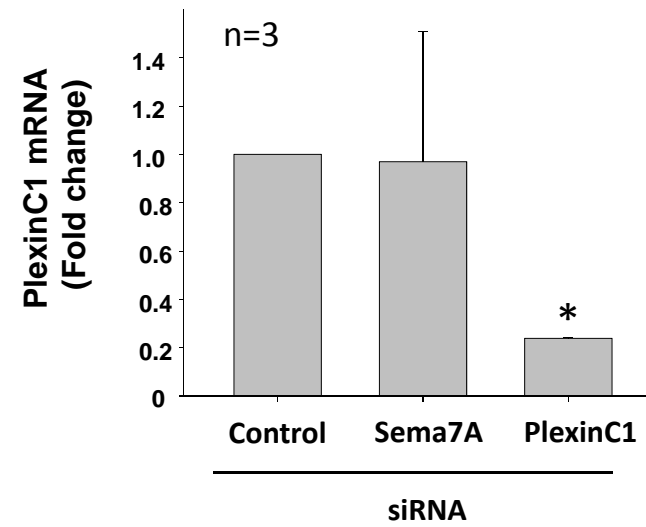

Supplement: S1 Fig — HLF were treated with siRNA (30 nM) for 48 h, and semaphorin-7A or plexin-C1 mRNA expression levels were analyzed by real-time PCR. Five and 3 experiments were analyzed for semaphorin-7A and plexin-C1, respectively. * indicates mRNA is decreased compared to the other siRNAs, with p<0.05. (PDF) [file pone.0170207.s001.pdf]

**Figure S2**

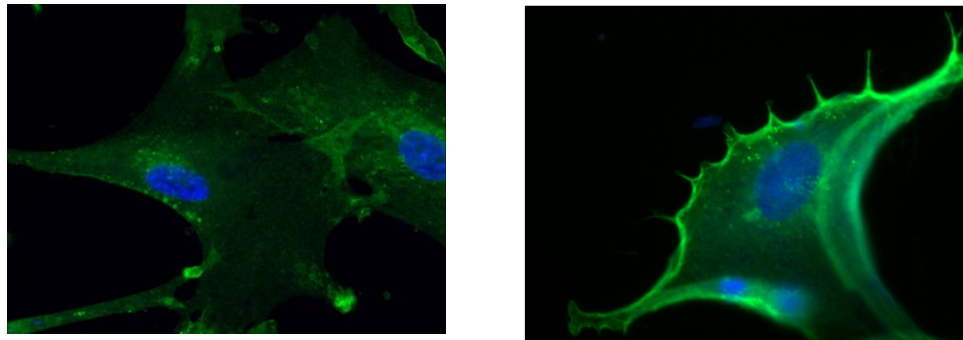

Supplement: S2 Fig — HLF were stained with a goat anti-semaphorin-7A antibody or a goat IgG control antibody. Immunofluorescent staining was observed with a 20x (left panel) or 60X objective (right panel). In all fibroblasts, diffuse membrane staining was apparent (left panel). In some fibroblasts, staining appeared to show localization of semaphorin-7A to cell edges, including structures resembling filipodia and lamellipodia. (PDF) [file pone.0170207.s002.pdf]

**Figure S3**

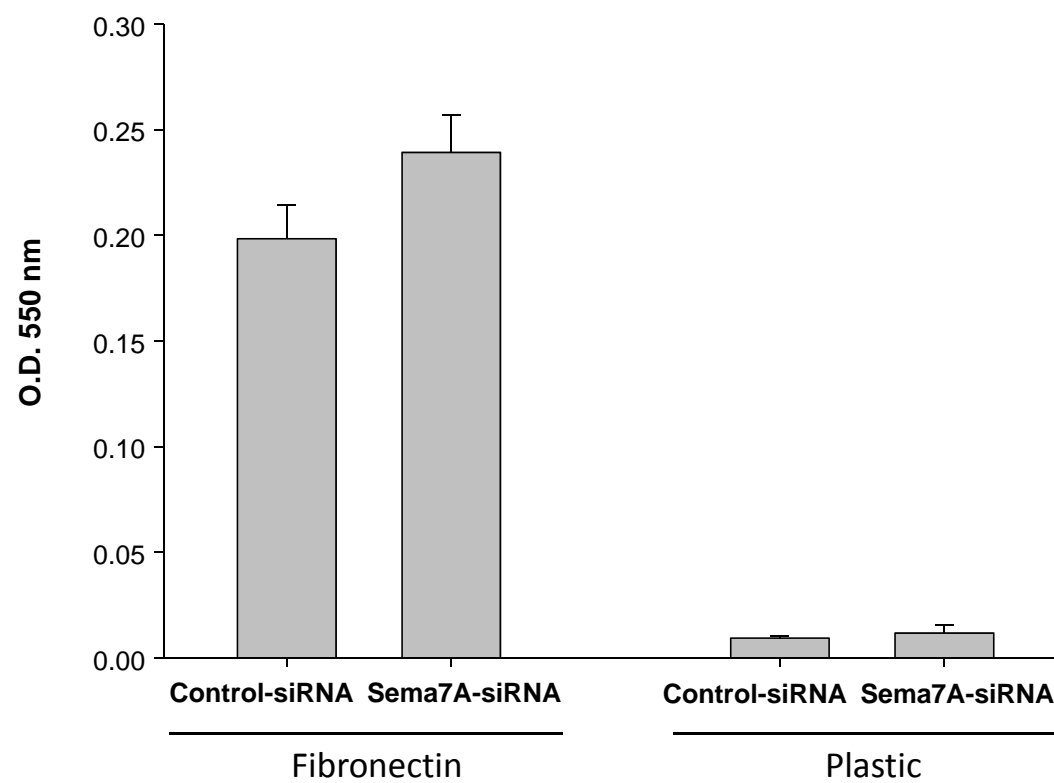

Supplement: S3 Fig — HLF were treated with the indicated siRNA for 48 h. HLF were resuspended in 0.1% BSA and were cultured for 30 min in plastic wells coated or not with fibronectin. The number of adherent cells was evaluated by crystal violet staining and optical absorption at 550 nm. Each condition was performed in quadruplicate and the graph shown is representative of two experiments. (PDF) [file pone.0170207.s003.pdf]

Figure S4 **New**

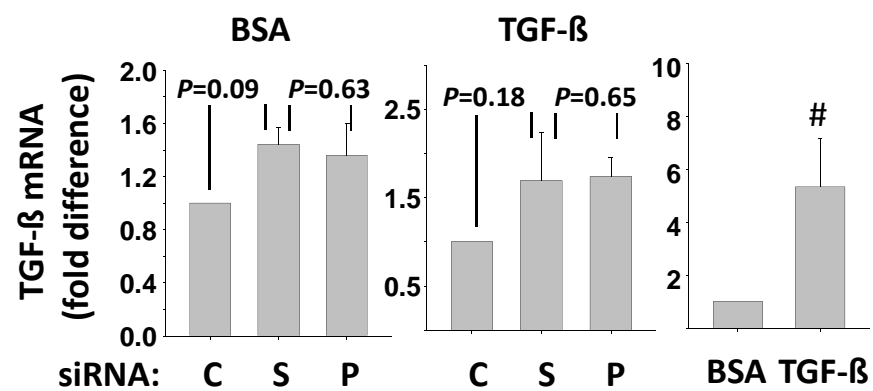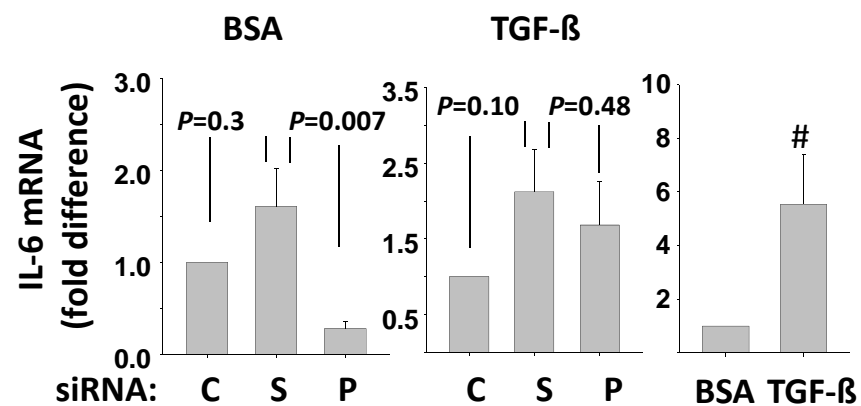

Supplement: S4 Fig — HLF were treated with control-siRNA (C), sema7A-siRNA (S) or plexin C1-siRNA (P) for 24 h before starvation in BSA for 24 h. Then, the cells were either kept in BSA or activated with TGF-ß (1 ng/ml) for 20 h. Real-time PCR was used to measure the level of expression of the indicated genes. For each gene, the first 2 graphs show the difference between C, S or P treatment, with C fixed at 1. The third graph displays the difference between TGF-ß and BSA after treatment with the control-siRNA. Graphs are an average of 3 experiments. P values from ANOVA analyses are shown. # indicates a statistical difference between TGF-ß and BSA. (PDF) [file pone.0170207.s004.pdf]

**Figure S5**

**A/**

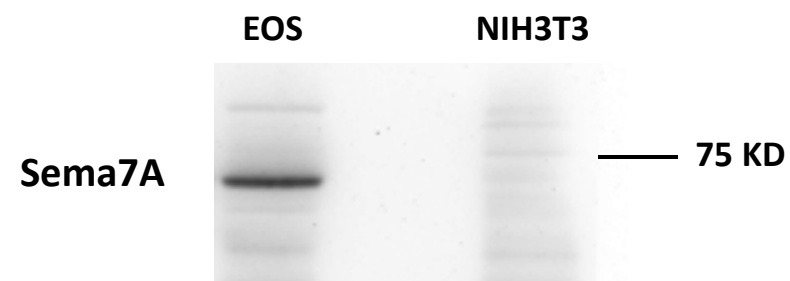

**B/**

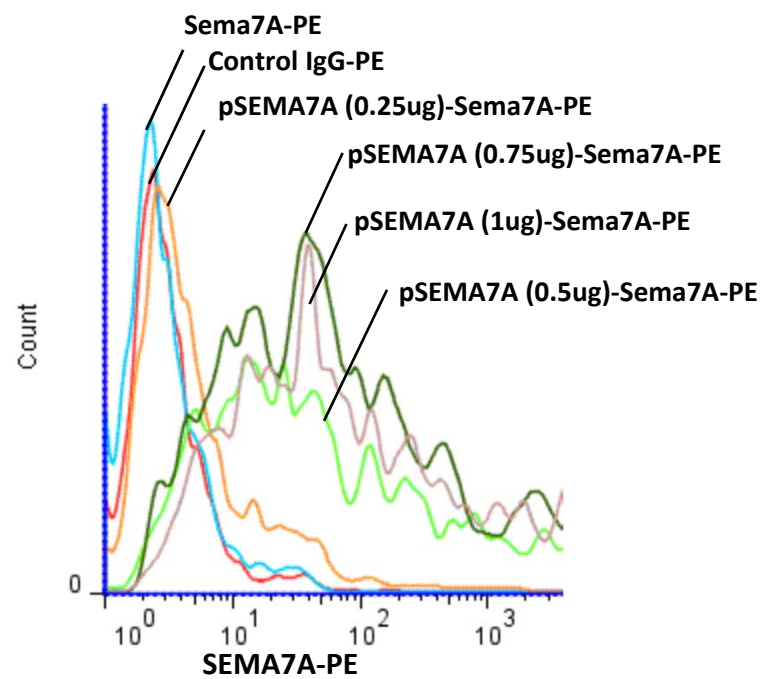

Supplement: S5 Fig — A/ The analysis of semaphorin-7A in the NIH3T3 fibroblast cell line by western-blot using an anti-human/mouse semaphorin-7A antibody, shows lack of semaphorin-7A in the cell line. Fresh eosinophils (EOS) were used as a positive control. B/ The expression of human semaphorin-7A in the NIH3T3 cell line was greatly induced after transfection with a semaphorin-7A expression vector. Flow cytometry analyses indicate that 0.75 μg of plasmid is optimal for expression of semaphorin-7A on the cell surface. (PDF) [file pone.0170207.s005.pdf]
