# Supplementary material for: Endogenous Semaphorin-7A Impedes Human Lung Fibroblast Differentiation
Source: PLoS One. 2017 Jan 17;12(1):e0170207. doi: 10.1371/journal.pone.0170207 (PMC5240965; doi:10.1371/journal.pone.0170207)
Supplement: S1 Table — (DOCX) [file pone.0170207.s006.docx]

**Supplemental Information**

**Endogenous Semaphorin-7A Impedes Human Lung Fibroblast Differentiation**

Esnault S.^1^, Torr EE.^1^, Bernau K.^1^, Johansson MW.^2^, Kelly EA^1^, Sandbo N.^1*^, Jarjour NN.^1*^

The University of Wisconsin-Madison School of Medicine and Public Health, Department of ^1^Medicine, Division of Allergy, Pulmonary and Critical Care Medicine, and ^2^Department of Biomolecular Chemistry, Madison, WI, USA

* These authors contributed equally as senior authors

**Supplemental Table 1: Primer sequences used for real-time PCR**

| **GENE** | **Forward primer** | **Reverse primer** |
| --- | --- | --- |
| **HUMAN** |  | |
| GUSB | CAGGACCTGCGCACAAGAG | TCGCACAGCTGGGGTAAG |
| SEMA7A | CTCCGCCCAGGGCCACCTAA | ACATGGCCTTTCCAGACGGCG |
| PLXNC1 | CCACCCCTTCACAGCTTGCGA | GCCACTGGACACTGTTCCTGGT |
| POSTN | TTATATGAGAATGGAAGGAATGAAAG | GTGCCATAAACATGGTCAATGG |
| FN1 | TCGAATTATGAGCAGGACCAGAA | CCTCCTCGAGTCTGAACCAAAA |
| COLA1A1 | CCAGAAGAACTGGTACATCAGCA | CGCCATACTCGAACTGGAAT |
| LAMB1 | GCCCGAACCCTACTGTATCG | TGGGAATTGCATATGAAGCATT |
| SRF | AGCACAGACCTCACGCAGA | GTTGTGGGCACGGATGAC |
| FOS | CTACCACTCACCCGCAGACT | AGGTCCGTGCAGAAGTCCT |
| TGFB1 | CGCGTGCTAATGGTGGAAA | TATACGACACACATGAGACGAACTTGAA |
| IL6 | TGCAGATGAGTACAAAAGTCCTGAT | CACCAATAACGTAGATCTAAGAAACG |
| **Mouse** |  | |
| Postn | Taqman Gene Expression Assay. Life Technologies Mm01284919_m1 | |
| Fn1 | AGACCATACCTGCCGAATGTAG | GAGAGCTTCCTGTCCTGTAGAG |
| Acta2 | AAACAGGAATACGACGAAG | CAGGAATGATTTGGAAAGGA |
